# Development of Surface-Coated Polylactic Acid/Polyhydroxyalkanoate (PLA/PHA) Nanocomposites

**DOI:** 10.3390/polym11030400

**Published:** 2019-03-01

**Authors:** J. J. Relinque, A. S. de León, J. Hernández-Saz, M. G. García-Romero, Francisco J. Navas-Martos, G. Morales-Cid, S. I. Molina

**Affiliations:** 1 Departamento de Ciencia de los Materiales e I.M. y Q. I., IMEYMAT, Facultad de Ciencias, Universidad de Cádiz, Campus Río San Pedro s/n, 11510 Puerto Real (Cádiz), Spain; manuelgerman.garciaro@alum.uca.es (M.G.G.-R.); sergio.molina@uca.es (S.I.M.); 2 Mechano(Bio)Chemistry, Max Planck Institute of Colloids and Interfaces, Science Park Postdam-Golm, 14424 Postdam, Germany; 3 Departamento de Ingeniería y Ciencia de los Materiales y del Transporte, Universidad de Sevilla, Camino de los Descubrimientos s/n, 41092 Sevilla, Spain; jhernandez32@us.es; 4 Centro Tecnológico del Plástico ANDALTEC, Ampliación Polígono Cañada de la Fuente, C/Vílchez s/n, 23600 Martos (Jaén), Spain; francisco-javier.navas@andaltec.org (F.J.N.-M.); gabrielmoralescid@gmail.com (G.M.-C.)

**Keywords:** polymer–matrix composites, ball milling, thermal properties, mechanical testing, physical methods of analysis

## Abstract

This work reports on the design and development of nanocomposites based on a polymeric matrix containing biodegradable Polylactic Acid (PLA) and Polyhydroxyalkanoate (PHA) coated with either Graphite NanoPlatelets (GNP) or silver nanoparticles (AgNP). Nanocomposites were obtained by mechanical mixing under mild conditions and low load contents (<0.10 wt %). This favours physical adhesion of the additives onto the polymer surface, while the polymeric bulk matrix remains unaffected. Nanocomposite characterisation was performed via optical and focused ion beam microscopy, proving these nanocomposites are selectively modified only on the surface, leaving bulk polymer unaffected. Processability of these materials was proven by the fabrication of samples via injection moulding and mechanical characterisation. Nanocomposites showed enhanced Young modulus and yield strength, as well as better thermal properties when compared with the unmodified polymer. In the case of AgNP coated nanocomposites, the surface was found to be optically active, as observed in the increase of the resolution of Raman spectra, acquired at least 10 times, proving these nanocomposites are promising candidates as surface enhanced Raman spectroscopy (SERS) substrates.

## 1. Introduction

Design of polymer nanocomposites by adding nano-sized particles has been widely performed since the early 1990s [1] in order to obtain new materials with enhanced mechanical, electrical, optical, thermal or magnetic properties using low amounts of loadings (typically up to 10 wt %) [2]. Nanocomposites are solid structures composed of at least two elements or phases where one has at least one feature in the nanometer scale (1–100 nm). Increase in the interface between nanofiller and polymer matrix enhances mechanical properties [3], while thermal or electric properties are dramatically modified when all nanofillers are in contact with each other and the percolation limit is reached [4]. Polymer nanocomposites consist of an organic continuous matrix (typically a commodity polymer) and one or more nanofillers. These nanofillers include silicates [5], nanocellulose crystals [6], metal-based nanoparticles [7] or carbon-based nanocomponents such as graphene or carbon nanotubes [8,9]. 

From a technological perspective, nanocomposites can be used as starting material in different industrial manufacturing techniques such as injection moulding, sintering, extrusion or hot pressing [10]. In the last years, there has been an increasing interest in the fabrication of filament, powder, and pellets for their further use in additive manufacturing processes [11]. However, a homogeneously distributed nanocomposite must be obtained prior to fabrication. Ball milling is one of the most effective methods to produce large amounts of material. On the one hand, advantages include its simplicity and versatility of production [12]. On the other hand, collision of the charge and balls can deliver high energy which causes plastic deformation under very high shear conditions. This provokes a decrease in the size of the material while energy is converted into heat. This heat leads to an increase of temperature inside the vessel which can cause polymer degradation, uncontrolled reactions or inhomogeneities in the particle size. Alternatives explored include milling under inert atmosphere or cryo-milling, although this increases the costs of the process [13,14]. 

Polylactic Acid (PLA) has emerged in the last decades as an interesting alternative from renewal resources to petroleum-based commodity polymers (Polyethylene, PE; Polypropylene, PP; Polystyrene, PS or Polyethylene Terephthalate, PET) because of its attractive mechanical properties, renewability, biodegradability, and relatively low cost [15]. PLA is nowadays present in different applications ranging from biomedicine to the packaging industry. However, PLA is too brittle for some applications [16]. Polyhydroxyalkanoate (PHA) presents a similar chemical composition and melting temperature than PLA although it presents poor processability. Nonetheless, when blended with PLA, it acts as a nucleating agent, improving the mechanical properties and barrier performance of the material, thus obtaining material with enhanced physical properties and processability [17]. PLA and derived materials are already being widely used in additive manufacturing processes, in particular in fused deposition modelling. Among other advantages, they are non-toxic and require less energy to be processed when compared to other commodity plastics such as PS or PP [18]. 

Graphite nanoplatelets (GNP) consist of stacked layers of 2D graphene sheets with a thickness of ~100 nm which represents a more economically viable alternative to graphene and other carbon-based nano-additives. Due to its layered conformation it is an easy-to-disperse nanofiller and can lead to enhanced structural and functional properties compared with the pristine polymer [19]. Increase in mechanical properties has been proven for PP [20] and epoxy resins [21] based composites when GNP is used. Likewise, it has been reported to increase thermal stability in a range of 40–80 °C in PE, Polyvinyl Alcohol, PVA or Polyanyline, PANI-based nanocomposites [22,23,24]. However, PLA-based nanocomposites designed so far do not exhibit much better thermal properties when GNP is used. An increase of 14 °C in thermal stability has been reported when 3 wt % GNP is used [25], while other results suggest no change in thermal properties [26]. Moreover, mechanical properties are not highly enhanced, even sometimes decrease above a certain content of nanofiller [27].

Silver nanoparticles (AgNP) are an interesting candidate among the inorganic nanoparticles as a filler. These nanoparticles have been mostly used in different biomedical applications due to its bactericidal properties [28,29,30]. Besides, surface plasmons of AgNP can be easily activated as a consequence of local excitation of the electric fields in their surroundings, which leads to more intense electronic transitions in molecules placed in the vicinity of the nanoparticles [31]. This effect can be used as an advantage to design materials with surface enhanced Raman scattering (SERS) with potential applications as sensors [32]. However, addition of AgNP to a PLA matrix has been reported to lead to a sensible decrease in mechanical properties. Alternatives so far reported include combining AgNP with other nanofillers that can reinforce the nanocomposite structure, although it increases the complexity of the material [33].

Herein, an alternative nanocomposite manufacturing method is reported, by milling the polymer and nanofillers under gentle shaking during short periods of time. Since the energy transferred was low, the shape and size of polymer remained intact, but the surface softened enough (T > T_g_) to allow the nano-additives to adhere. This led to fabrication of nanocomposites in the shape of thin-coated pellets. Processability of these pellets was further proved by preparing testing samples via injection moulding, and the mechanical and thermal properties were evaluated. The aim of the work was to prove that surface selective, small addition of nano-additives affects the polymer matrix in a different manner, than if they were in a homogeneous bulk distribution, and can lead to functional materials with enhanced properties.

## 2. Materials and Methods

### 2.1. Materials

Polylactic acid/PHA pellets (20 wt % PHA, M_w_ = 4 × 10^5^ g/mol) were supplied by Colorfabb. Graphite nanoplatelets (GNP, 2 µm × 5 µm × 10 nm) were supplied by Avanzare (Navarrete, Spain).

Silver nanoparticles (AgNP) were synthesized as described in [34]. Briefly, AgNO3 and polyvinlyl alcohol were dissolved in an alcoholic solution and then exposed to microwave radiation. After 30 s, AgNP were synthesized. Then, nanoparticles were filtered and dried before storage.

Synthesis of nanocomposites was carried out by mechanical mixing using a planetary ball mill Retsch PM-100. PLA/PHA polymer matrix and nano-additives (concentrations ranged from 0.05 to 0.10 wt %) were mixed and shaken during 10 min at 400 rpm. 

Pellets were then dried at 80 °C for 3 h before the injection to avoid water absorption, since PLA/PHA is hygroscopic. Samples for mechanical testing were prepared by injection moulding at 6 bar in a pneumatic machine Ray Ran RR/TSMP (Nuneaton, UK). A summary of the different nanocomposites together with injection temperature conditions is depicted in Table 1.

### 2.2. Characterisation

Optical micrographs were taken using a Nikon Eclipse MA100 (Tokyo, Japan) inverted light microscope. Images were taken using a 50× objective. Image analysis was performed using the free image analysis software ImageJ (http://rsb.info.nih.gov/ij/).

High resolution transmission electron microscopy (HRTEM) images of the synthesized AgNP were taken with a JEOL 2100 (Tokyo, Japan) equipped with a LaB6 electron source working at 200 kV. Image analysis was performed using the free software JMicrovision. 

The internal structure of the nanocomposites was obtained by cross-section imaging using ion-induced secondary electrons (ISEs), of a FEI QUANTA 200 3D DUAL FIB/SEM (Hillsboro, OR, USA) focused ion beam-scanning electron microscope (FIB-SEM). Samples were C sputtered in a Balzers SCD 004 Sputter Coater (Balzers, Liechtenstein) prior to scanning to reduce sample charging. A platinum layer was deposited on the surface of the sample to protect the area of interest during subsequent milling processes. Then, trenches were milled on both sides of metal layer by FIB (Ga^+^ ions) and a cross-section was revealed by ISEs. Chemical element identification was performed by an EDX microanalyzer coupled to a FEI QUANTA 200 SEM instrument (Hillsboro, OR, USA).

Chemical composition of the nanocomposites was determined using a confocal Raman microscope (alpha300, WITec, Ulm, Germany). The microscope was equipped with a piezo scanner (P-500, Physik Instrumente) and a 50× objective (Nikon, NA 0.6, Tokyo, Japan). A linearly polarized laser (λ = 532 nm, power output 50 mM) was focused onto the sample and the Raman scattered light was detected on a thermoelectrically cooled charge-coupled device detector (DU401A-BV, Andor, Belfast, UK) with an integration time of 2 s. Spectra from at least five different positions on the sample were collected. Samples for mechanical characterisation were prepared and tested according to ISO 527-1 and ISO 527-2 (tensile testing, Universal Testing Machine Tinius Olsen 10 KS, Horsham, PA, USA), ISO 180 (impact testing, Impact tester Metrotec IMPATS 15, Lezo, Spain), and ISO 306 (Vicat testing, ATS Faar MP-3, Milan, Italy). At least 5 samples were measured in all cases (except for Vicat testing, where 3 samples were tested, according to the standard) and results were averaged. Differential scanning calorimetry (DSC) (Mettler-Toledo 822e, Columbus, OH, USA) was performed to investigate the thermal properties of the nanocomposites. Temperature sweeps were performed from 30 to 200 °C at a rate of 10 °C/min.

## 3. Results and Discussion

### 3.1. Structural Characterisation

**Figure 1:**
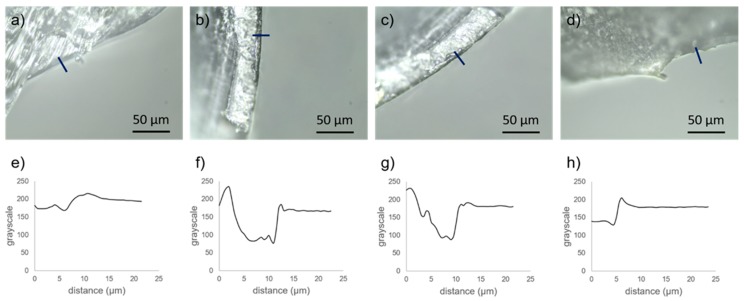
Optical micrographs of (**a**) PLA/PHA; (**b**) PLA/PHA+GNP0.05; (**c**) PLA/PHA+GNP; (**d**) PLA/PHA+AgNP. Blue lines depict the contrast profiles of pellets surface plotted for (**e**) PLA/PHA; (**f**) PLA/PHA+GNP0.05; (**g**) PLA/PHA+GNP; (**h**) PLA/PHA+AgNP. (PLA: Polylactic Acid; PHA: Polyhydroxyalkanoate; GNP: Graphite NanoPlatelets; AgNP: Silver nanoparticles).

Nanocomposite pellets were prepared using a planetary ball mill. PLA/PHA pellets were introduced together either with GNP or AgNP inside the milling vessel and shaken at 400 rpm for 10 min. The pellet size did not seem to vary after the milling procedure. However, a clear change in the surface colour was appreciated in all cases to the naked eye. In the case of PLA/PHA+GNP0.05 and PLA/PHA+GNP black-coloured pellets were obtained while a light green shade was observed in the case of PLA/PHA+AgNP composites. Pellets were mechanically cut and their cross-section was observed under an optical microscope (Figure 1a–d). 

**Figure 2:**
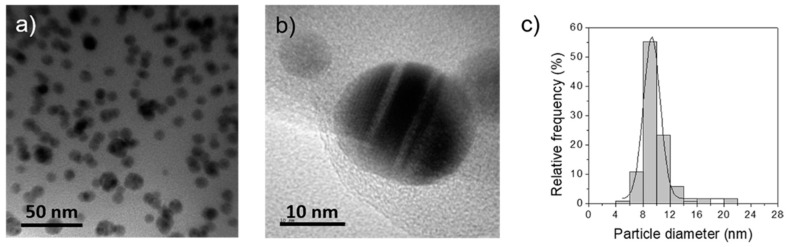
(**a**,**b**) Transmission electron microscopy images and (**c**) histogram depicting average particle distribution of AgNP. Average particle diameter, D_p_ = 9.9 ± 2.2 nm.

While no dramatic change in contrast was observed on the surface of PLA/PHA (Figure 1a) pellets, a thin, black layer could be appreciated on the surface of PLA/PHA+GNP0.05 (Figure 1b) and PLA/PHA+GNP (Figure 1c) pellets. However, inner part of the pellets did not show any significant change, which seems to indicate only the surface was altered. A contrast profile of the micrographs is shown for clearer interpretation (Figure 1e–h). A decay in the contrast of approximately 10 μm right at the surface of the pellets was observed for PLA/PHA+GNP0.05 (Figure 1f) and PLA/PHA+GNP (Figure 1g). This size is well in agreement with the dimensions of GNP (2 µm × 5 µm × 10 nm), which seems to indicate that the nanoplatelets were adhered onto the surface. Further analyses by optical microscopy showed that 0.1 wt % GNP yielded to a full coverage all over the PLA/PHA, while 0.05 wt % GNP composites presented some areas which were non-coated. For this reason, 0.1 wt % was selected as the optimal concentration for a homogeneous coating and it was used to prepare the AgNP nanocomposite. Therefore, it can be concluded that 0.10 wt % is a sufficiently high concentration to have homogeneous surface coverage of the PLA/PHA nanocomposites. In the case of PLA/PHA+AgNP (Figure 1d), the slight glow observed on the surface can be associated to the fact that these AgNP possess an absorption band in the visible range [34]. Unlike GNP, the size of AgNP (D_p_ = 9.9 ± 2.2 nm, Figure 2) is below the diffraction limit of an optical microscope (200 nm) and is not possible to determine if multilayers or only a monolayer of AgNP is formed onto the PLA/PHA surface. Nanocomposite synthesis was then repeated by increasing the milling process up to 20 min to check if longer times led to a different nanofiller distribution. However, no significant differences were observed by optical microscopy. These results suggest that the milling process is not strong enough to grind up the PLA/PHA pellets, but energy transmitted is high enough to increase PLA/PHA temperature above T_g_ (55–60 °C) [35]. This would increase their plasticity favouring physical adsorption of either GNP or AgNP onto their surface. 

**Figure 3:**
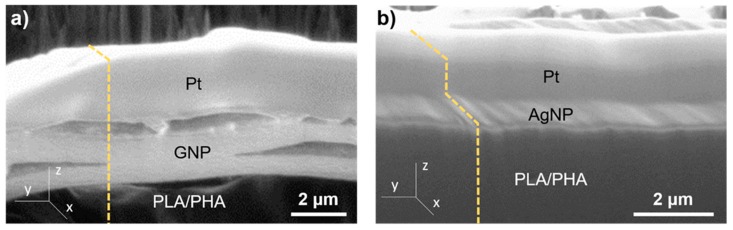
Induced Secondary Electron micrographs of (**a**) PLA/PHA+GNP and (**b**) PLA/PHA+AgNP. Dashed, yellow lines depict the profile of the cross-section in the *z*-axis.

To gain further insight and prove this hypothesis, structural characterisation was carried out via FIB milling and SEM observation. The FIB-SEM equipment allowed for in-situ generation and observation of cross-sections of materials with submicrometric resolution. Careful optimization must be considered when working with soft matter (i.e., polymers) since they can be easily damaged by the high doses of the Ga^+^ beams and electrons due to its non-conductive nature [36,37]. Image conditions were tuned well enough to see this contrast and discern between the PLA/PHA matrix and GNP. Figure 3 shows the profile of a cross-section of the nanocomposites fabricated. 

**Figure 4:**
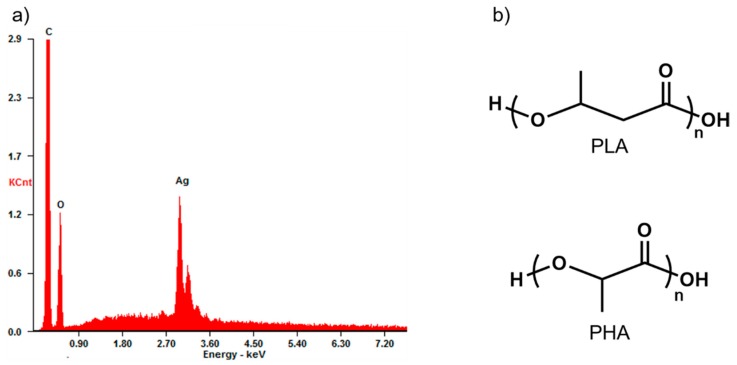
**(a**) EDX spectrum of PLA/PHA+AgNP; (**b**) chemical structure of PLA and PHA.

A difference of contrasts on the top layer of the pellets after cross-section milling was observed. In Figure 3a contrast of 1–2 layers of ~2 μm thick was observed. These sizes are in good agreement with stacked layers of GNP. In a similar fashion, Figure 3b shows a ~60 nm thickness, homogeneous layer with a lighter contrast on top of the surface of the pellet, which can be attributed to the AgNP. Complementary EDX measurements were performed in this case (Figure 4a) to study the chemical composition of the surface. C and O peaks correspond to the signal from PLA/PHA matrix, as depicted in Figure 4b. Moreover, a strong Ag peak can be observed clearly evidencing the presence of AgNP in the surface. Since Ag is a much heavier atom than C and O, it can be assumed that it is normal to observe such high intensity for this peak, even when PLA/PHA+AgNP contains only 0.1 wt % of silver. This is further proof of what was observed by optical microscopy, and so, nanocomposites selectively modified only on the surface were successfully produced.

### 3.2. Mechanical Properties

**Figure 5:**
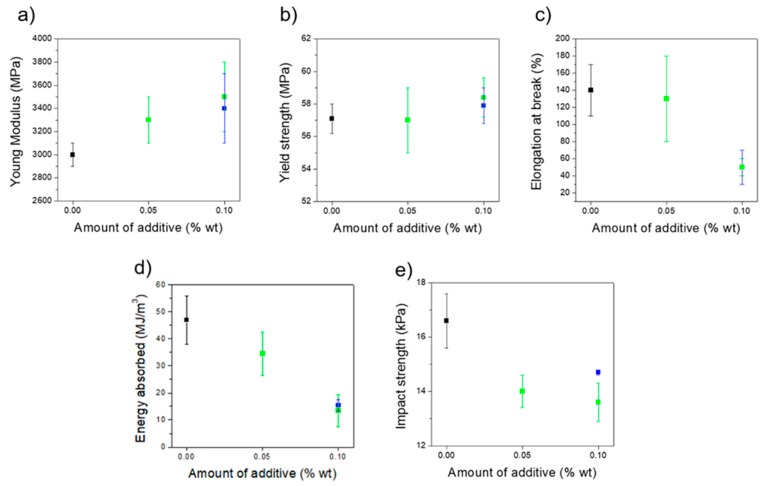
(**a**) Young modulus; (**b**) yield strength; (**c**) elongation at break, (**d**) energy absorbed from tensile experiments; and (**e**) impact strength of unmodified PLA/PHA (black), PLA/PHA+GNP (green), and PLA/PHA+AgNP (blue). Results are shown as a function of the amount of nano-additive used in the composite.

Samples were prepared by injection moulding and tensile and impact tests were performed to study the influence of the addition of GNP or AgNP in the mechanical properties. Tensile testing results show an increase of 10% and 2% in the Young modulus and yield strength, respectively, when PLA/PHA was loaded either with GNP or AgNP (Figure 5a,b). As expected, the presence of a nano-additive increases the elastic mechanical properties, when compared to the unmodified polymer. Even if this increase was not very high due to the low concentration of additive, these results are particularly interesting in the case of PLA/PHA+AgNP, where a decrease in these values was previously reported [33]. However, a significant decrease from 140% to 50% strain was also observed in the elongation at break for both composites when a concentration of 0.10 wt % was used (Figure 5c), proving that nanocomposites are more fragile than unmodified PLA/PHA. Energy absorbed in the tensile test and impact strength (Figure 5d,e) also show the nanocomposites are weaker than PLA/PHA. A smaller decrease in the impact strength was observed for PLA/PHA+AgNP than for PLA/PHA+GNP. The smaller size of AgNP (10 nm) may allow them to move easily along the material together with the PLA/PHA chains to dissipate the energy of an impact. Conversely, GNP possesses two dimensions in the micrometric scale (2 μm × 5μm × 10 nm), which probably causes more hindrance to reorganize during an impact, leading to lower impact strength values. Other authors studied the influence of GNP on PLA (not PLA/PHA blends) and embrittlement of material is always reported. Botta et al. [26] and Gao et al. [38] reported a minimum decrease of elongation at break of 5% and 11% strain, respectively, for GNP concentrations of 5 wt %. However, in both cases unmodified PLA did not possess higher elongation at break. In these cases, 50–60 MPa yield strengths were reported, similar to the exhibited on this work. Gao et al. [38] also reported a linear decrease of energy absorbed with GNP content. In their case, maximum energy absorbed of 4.7 MJ/m3 was achieved when PLA was loaded with 5 wt % GNP, while nanocomposites herein designed can absorb energies above 10 MJ/m3 in all cases. Pinto et al. [27] reported high elongation at break of above 200% strain, with 0.4 wt % GNP concentration. However, yield strength values reported in this case were very low (30 MPa). Molecular weight of PLA used in their study was sensibly lower (105 g/mol) than in the present work (4 × 10^5^ g/mol), which makes PLA more deformable but weaker. It can be hypothesized that presence of PHA makes the polymer matrix more plastic while at the same time keeps good elastic mechanical properties than pure PLA. Thus, it can be stated that the nanocomposites developed in this work present relatively high elongations at break, can absorb reasonably high amounts of energy, and possess enhanced strength and elastic modulus.

### 3.3. Thermal Properties

**Figure 6:**
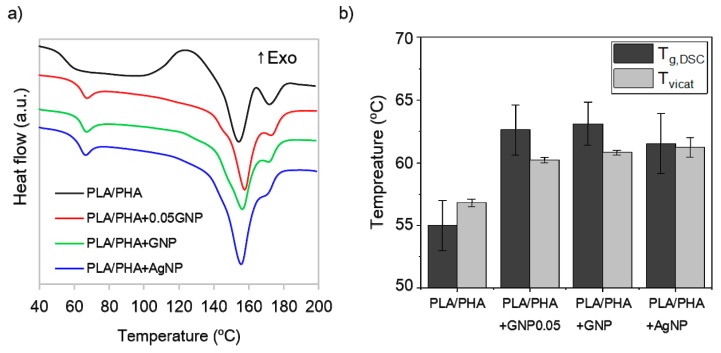
(**a**) DSC thermograms of PLA/PHA (black); PLA/PHA+0.05GNP (red); PLA/PHA+GNP (green), and PLA/PHA+AgNP (blue); (**b**) comparison between extracted T_g_ from DSC sweeps (dark grey bars) and softening temperature from Vicat assay (light grey bars). (DSC: Differential Scanning Calorimetry).

Thermal stability of the nanocomposites was evaluated. For this purpose, differential scanning calorimetry curves of the as-prepared nanocomposites were compared with Vicat testing of different probes prepared by injection moulding. The Vicat softening point was estimated as the temperature when a sample is penetrated by a 1 mm × 1 mm flat needle, and it can be assumed as the maximum temperature of the use of a material. The obtained DSC curves (Figure 6a) show an increase of 7–8 °C in T_g_ when nanocomposites are compared to unmodified PLA/PHA. When compared, these values match reasonably well with measured Vicat softening temperatures (Figure 6b). Other authors claim a light increase of 3 °C [38] or even no changes [26] when 5 wt % GNP is added to a PLA matrix, while a decrease in thermal stability values were reported when 1 wt % AgNP was used [33]. Surface-coated nanocomposites herein reported show enhanced thermal stability with only 0.1 wt % regardless of the nano-additive used. Admitting this low content is below the percolation limit, typically around 5 wt % [19], a non-homogeneous distribution of the additives might locally enhance heat transfer in the nanocomposites, conferring them higher thermal stability. Thus, it can be stated that this fabrication method leads to nanocomposites with enhanced thermal properties, in view of the obtained results. Since the nano-additives concentration used was very low, hopefully this can be a potential method to design nanocomposites in a more cost-efficient way. 

### 3.4. Raman Characterisation

**Figure 7:**
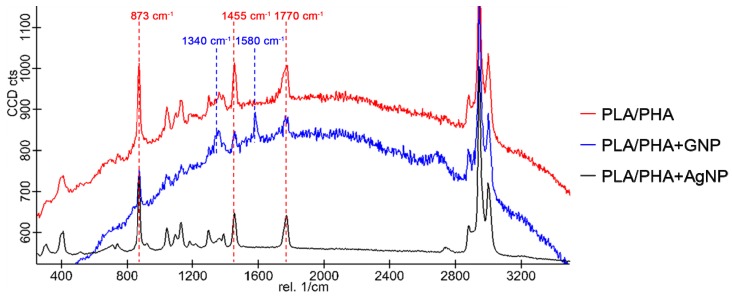
Raman spectra of PLA/PHA (**red**); PLA/PHA+GNP (**blue**); and PLA/PHA+AgNP (**black**). Characteristic peaks of PLA/PHA corresponding to νC–OO and δCH_3_ asymmetric modes and C=O stretching at 873, 1455, and 1770 cm^−1^, respectively, are indicated in red. Characteristic D and G peaks of GNP at 1340 and 1580 cm^−1^, respectively, are indicated in blue. All spectra were taken with an integration time τ_i_ = 2 s.

Nanocomposites were studied via Raman spectroscopy. Figure 7 shows spectra of PLA/PHA, PLA/PHA+GNP and PLA/PHA+AgNP nanocomposites. 

Characteristic peaks for PLA/PHA can be observed in all cases at 873, 1455, and 1770 cm^−1^ corresponding to νC–COO and δCH_3_ asymmetric modes and C=O stretching, respectively (see Figure 4b for PLA and PHA molecular structures). PLA/PHA+GNP also exhibited another band at 1580 cm^−1^ which corresponded to the G peak of GNP [26]. It can also be observed that the background corresponding to fluorescence scattering of the sample was non-negligible for PLA/PHA and PLA/PHA+GNP samples. This fluorescence scattering is a competitive process with Raman and its presence depends on the sample and the type of laser used to excite the sample. Fluorescence background can be very high and decreases the quality of the Raman spectrum acquired. Typical processes to avoid this include to emit a high intensity, short pulse with the Raman laser [39]. However, this is not needed when noble metal nanoparticles are used since they are also able to quench fluorescence scattering themselves [40,41]. This is in well agreement with the obtained results, where fluorescence is completely eliminated in the case of PLA/PHA+AgNP nanocomposites. To ensure the excitation and relaxation of the electrons on surface is the same (i.e., Raman spectra are comparable), the acquisition was performed by keeping integration times constant down to 2 s. This was done in order to i) avoid auto-bleaching of the fluorescence background due to high exposure times with the laser, and ii) compare the signal-to-noise ratio of the different spectra to check their quality at low integration times. In this way, it could be assured that the noise of the acquired spectra decreased sensibly caused exclusively by the presence of the AgNP on the surface. These spectra were taken in different points of the surface of PLA/PHA+AgNP to check the homogeneous distribution of the AgNP and the absence of fluorescence background throughout the whole surface. 

As proof of concept, Table 2 shows the increase of signal-to-noise ratio (SNR) for the three characteristic peaks of PLA/PHA, indicating an improvement of ~10 times in the resolution of the spectra acquired when PLA/PHA+AgNP nanocomposite was used. These results not only provide evidence that these nanocomposites present AgNP homogeneously distributed on the surface but also show that these nanocomposites are promising candidates for large surface enhanced Raman spectroscopy (SERS) devices [42], due to high availability and easy processability of the PLA/PHA matrix, and the low necessary amount of silver needed.

## 4. Conclusions

In conclusion, a new technique has been developed to manufacture nanocomposites by mechanical milling under mild conditions. This led to surface modification of polymer pellets with different nanomaterials (e.g., GNP and AgNP). Advanced microscopic characterisation showed homogeneous surface distribution of both GNP and AgNP. The designed nanocomposites exhibited enhanced mechanical and thermal properties. In particular, Young modulus and strength increased due to either GNP or AgNP, while PLA/PHA matrix enhanced impact resistance and energy absorption when compared to PLA-based nanocomposites. This was particularly remarkable in the case of AgNP, where as far as it is known, this is the first report where mechanical properties are enhanced. Thus, PLA/PHA+AgNP revealed interesting optical properties (an increase of ~10 times in the SNR) due to the plasmon activation of AgNP in surface, which makes it a potential good candidate for SERS-based sensing devices. The combination of these properties together with this new fabrication process will open a new lead on the design of processable materials where additives are valuable and small amounts are preferably used, but functional materials are required in large scales.

## 5. Patents

The synthesis procedure to obtain nanocomposites depicted in this work was patented. The patent number is WO/2015/173439 A1 and was granted on 19 November 2015.

## Figures and Tables

**Table 1:** Prepared Polylactic acid (PLA)/Polyhydroxyalkanoate (PHA) nanocomposites and their main synthesis characteristics.

Material	Nanofiller	Additive Concentration wt %	Processing T (Cylinder/Mould) °C
PLA/PHA	-	-	192/70
PLA/PHA+GNP0.05	GNP	0.05	186/72
PLA/PHA+GNP	GNP	0.10	186/72
PLA/PHA+AgNP	AgNP	0.10	193/76

**Table 2:** Signal-to-noise ratios (SNRs) for PLA/PHA characteristic Raman peaks for PLA/PHA matrix and PLA/PHA+AgNP nanocomposite. Δ(SNR) depicts the increase in the SNR when the silver is on the surface.

Raman Peak (cm^−1^)	SNR (PLA/PHA)	SNR (PLA/PHA+AgNP)	Δ (SNR)
873	7.6	88	11.58
1455	4.2	40	9.52
1770	3.1	38	12.26
